# Phenylthiourea Binding to Human Tyrosinase-Related Protein 1

**DOI:** 10.3390/ijms21030915

**Published:** 2020-01-30

**Authors:** Xuelei Lai, Harry J. Wichers, Montserrat Soler-López, Bauke W. Dijkstra

**Affiliations:** 1 Laboratory of Biophysical Chemistry, University of Groningen, 9717 GA Groningen, The Netherlands; 2 European Synchrotron Radiation Facility, 38043 Grenoble, France; 3 Wageningen University & Research, 6708 WG Wageningen, The Netherlands

**Keywords:** human tyrosinase, human tyrosinase-related protein, phenylthiourea, inhibitor, crystal structure, *N*-glycosylation, albinism, melanogenesis, zinc–copper enzymes

## Abstract

Tyrosinase-related protein 1 (TYRP1) is one of the three human melanogenic enzymes involved in the biosynthesis of melanin, a pigment responsible for the color of the skin, hair, and eyes. It shares high sequence identity with tyrosinase, but has two zinc ions in its active site rather than two copper ions as in tyrosinase. Typical tyrosinase inhibitors do not directly coordinate to the zinc ions of TYRP1. Here, we show, from an X-ray crystal structure determination, that phenylthiourea, a highly potent tyrosinase inhibitor, does neither coordinate the active site zinc ions, but binds differently from other structurally characterized TYRP1-inhibitor complexes. Its aromatic ring is directed outwards from the active site, apparently as a result from the absence of polar oxygen substituents that can take the position of water molecules bound in the active site. The compound binds via hydrophobic interactions, thereby blocking substrate access to the active site.

## 1. Introduction

The human melanogenic enzymes tyrosinase (TYR), tyrosinase-related protein 1 (TYRP1), and tyrosinase-related protein 2 (TYRP2) take part in the biosynthesis of melanin, a pigment that is responsible for the color of the skin, hair, and eyes [1]. Mutations of the genes that code for these proteins may lead to loss of skin pigmentation, which in turn has been linked to an increased incidence of carcinoma [2]. In addition, loss of melanin is often (but not always) associated with abnormal development of the retina, which causes severe visual defects [3]. The precise relation between melanin biosynthesis and eye development has not yet been established, although the tyrosine hydroxylation product L-3,4-dihydroxyphenylalanine (L-DOPA) has been hypothesized to serve as the link [3].

Although the role of TYR in the melanin biosynthetic pathway is generally accepted, the function of TYRP1 is less clear [1]. The crystal structure of the intra-melanosomal domain of TYRP1 showed that the active site contains two zinc ions, bound virtually indistinguishably from the binuclear type 3 copper site of bacterial and fungal tyrosinases [4]. Because zinc ions, in contrast to copper ions, are not redox-active, this implies that TYRP1 must have a different activity from TYR, as indeed corroborated experimentally [4]. In this respect, the 5,6-dihydroxyindole-2-carboxylic acid (DHICA) oxidase activity of the recombinant melanosomal domain of human TYRP1, reported recently [5], may result from the presence of (trace amounts of) copper ions, and further research is needed to establish the physiological significance of this observation.

Binding of L-tyrosine and the L-DOPA analogue L-mimosine to TYRP1 showed that these compounds do not directly interact with the zinc ions of TYRP1, in contrast to, for example, the tyrosine analogue tyrosol, which directly coordinates Cu-A in *B. megaterium* tyrosinase [6]. To further investigate the binding of inhibitors to melanogenic enzymes, we determined the interaction of TYRP1 with the inhibitor phenylthiourea (PTU), the most potent inhibitor of human TYR that we tested. To reduce any effect of amino acid differences, we used a TYRP1 triple mutant (TYRP1-3M) in which the three non-conserved active site residues were replaced by the corresponding ones of TYR (Y362F/R374S/T391V). The activity of this triple mutant is similar to that of native TYRP1, both in the Zn- and Cu-substituted forms; replacing the zinc ions by copper ions confers DHICA oxidase activity to both TYRP1 and the 3M mutant [4]. Our results show that PTU does not coordinate the active site zinc ions, but has a very different binding mode compared to the other tyrosinase inhibitors bound to TYRP1. Most conspicuously, the aromatic ring of PTU is directed outwards from the active site, which appears to result from the absence of polar oxygen substituents that can take the position of water molecules bound in the active site. The compound binds via hydrophobic interactions, thereby blocking substrate access to the active site.

TYR, TYRP1, and TYRP2 not only differ in their enzymatic activity, but also in their *N*-glycosylation patterns. Three *N*-glycosylation sequons (Asn86, Asn290, Asn371 (TYR sequence numbering)) are conserved in the human melanogenic enzymes. No disease-related mutations are known for TYR Asn86 and Asn290, suggesting that glycosylation of these sites is not essential for functionality. In contrast, Asn371 mutations have been found in patients with classic tyrosinase-dependent oculocutaneous albinism. Asn371 is located at the rim of the entrance to the active site pocket, about 15–20 Å from the TYR copper ions, suggesting that the absence of glycosylation at this site severely compromises the enzyme’s activity, or stability, or both.

## 2. Results

### 2.1. Inhibition of Human TYR by Phenylthiourea (PTU)

**Figure 1:**
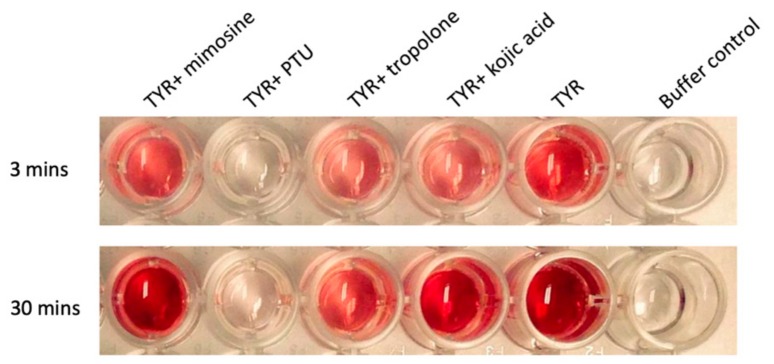
Colorimetric tyrosinase activity assay showing the inhibition by different inhibitors. The images were taken at 3 min (upper panel) and 30 min (lower panel) after the start of the reaction. The buffer control contained 0.5 mM 3-methyl-2-benzothiazolinone hydrazine hydrochloride hydrate (MBTH) and 2 mM L-3,4-dihydroxyphenylalanine (L-DOPA) in 40 mM phosphate buffer, pH 6.5. The other wells contained the buffer control condition with added 0.4 μM tyrosinase (TYR) protein and 2 mM inhibitor, as indicated in the figure. PTU: phenylthiourea.

Various compounds with well-known reductive tyrosinase activity were tested in an on-plate colorimetric assay for their potency against the melanosomal domain of TYR. Interestingly, these experiments demonstrated that PTU is the most potent inhibitor of human tyrosinase (Figure 1), similar to previous studies with mushroom tyrosinase [7]. PTU has been suggested to adopt an unusual bridging binding mode when interacting with a binuclear copper(II) center [8]. Moreover, no crystal structures of tyrosinase with bound PTU are available in the Protein Data Bank (PDB). Therefore, it was deemed of interest to investigate the structural details of the interaction of PTU with TYRP1-3M, a triple mutant of TYRP1 in which the three non-conserved active site residues were replaced by the corresponding ones of TYR (Y362F/R374S/T391V) [4].

### 2.2. Interaction of Phenylthiourea (PTU) with TYRP1-3M

**Figure 2:**
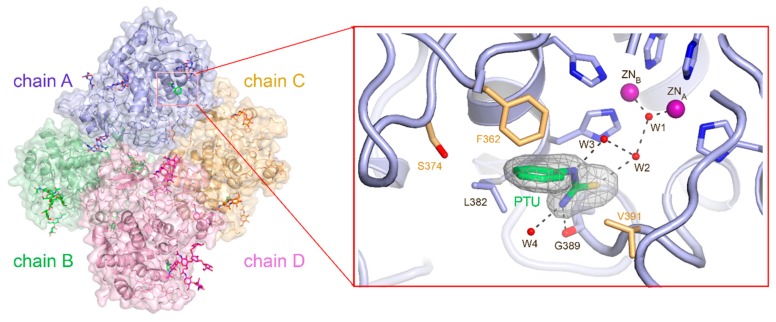
Tyrosinase-related protein 1 triple mutant (TYRP1-3M) with PTU bound in the active site. The TYRP1-3M crystal contained four molecules in the asymmetric unit (chains A, B, C, and D); N-linked sugars are shown as sticks in magenta; PTU from chain A is shown as sticks in blue at the active site. The details of the PTU interaction network in the active site is shown in the blow-up; W1, W2, W3, and W4 correspond to water molecules 15, 9, 331, and 356, respectively, in Protein Data Bank (PDB) 5M8S. PTU is mainly stabilized by hydrophobic interactions with the protein. The three residues mutated in the 3M mutant are shown in gold (F362, S374, V391).

The crystal structure of TYRP1-3M with bound PTU was elucidated at 2.2 Å resolution (Figure 2; Table 1). The crystal was formed with four molecules in the asymmetric unit (chains A, B, C, and D) (Figure 2), identical to native TYRP1 [4]. The PTU aromatic ring pointed outwards from the binuclear zinc site; its position was stabilized by hydrophobic interactions with the side chains of Phe362, Leu382, and Val391 (Figure 2). The thiourea amino group was hydrogen bonded to the backbone oxygen of Gly389 and a water molecule (W4), whereas the amide nitrogen and thiourea sulfur had hydrogen bonds with water molecules W3 and W2, respectively (Figure 2). Surprisingly, PTU did not interact with the binuclear metal site, but blocked substrate access to the active site. All interactions that stabilized PTU in TYRP1-3M were conserved in the TYR active site, which suggests that PTU can bind in a similar way to native TYR.

**Figure 3:**
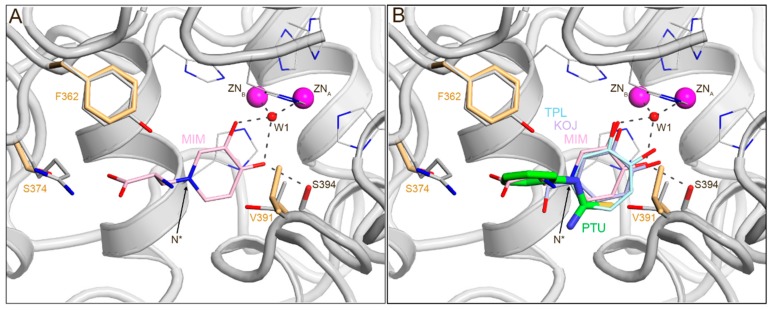
PTU binds in the active site of TYRP1-3M with its aromatic ring pointing outwards from the binuclear metal center, whereas the other inhibitors point inwards. (**A**) Binding of the L-DOPA analogue mimosine (cartoon atoms colored in light pink; PDB 5M8R); N* indicates the position of the nitrogen atom in the aromatic ring of mimosine. (**B**) Superimposition of TYRP1-3M with bound mimosine (MIM, light pink; PDB 5M8R), tropolone (TPL, light blue; PDB 5M8T), kojic acid (KOJ, gray; PDB 5M8Q), and PTU (PTU, green; PDB 5M8S). MIM, TPL, and KOJ bind with their aromatic ring pointing inwards, towards the binuclear metal site. PTU binds outward with its aromatic ring. In the figure, oxygen atoms are colored in red, and nitrogen atoms in blue; zinc atoms are labeled as ZN_A_ and ZN_B_; W1 is the bridging water molecule between ZnA and ZnB.

Superimposition of the PTU-inhibited TYRP1-3M structure and TYRP1-3M structures with bound kojic acid, mimosine, and tropolone (PDB entries 5M8Q, 5M8R, and 5M8T, respectively [4]) revealed that PTU binds very differently from these compounds (Figure 3). Most conspicuously, the aromatic ring of PTU was directed outwards from the active site, whereas the other inhibitors had their ring system oriented towards the binuclear metal-binding site. The amide nitrogen (Figure 3B, annotated as N*) of PTU perfectly superimposed on the ring C5 carbon atom of kojic acid, the ring nitrogen atom of mimosine, and the Cα5 carbon atom of tropolone (Figure 3). The space occupied by water molecules W2 and W3 in the PTU-bound structure was taken by the ring oxygen atoms of kojic acid, mimosine, and tropolone. These oxygen atoms were hydrogen bonded to the bridging water molecule W1 between the two metal ions in the active site (Figure 3). The PTU sulfur atom coincided with a ring carbon atom of the inhibitors (e.g., the Cε atom of the mimosine ring). Thus, it appeared that the different binding mode of PTU originated from the absence of polar oxygen substituents that could take the position of bound water molecules in the active site.

## 3. Discussion

The precise function and activity of TYRP1 has been puzzling [1,4]. Initial reports suggested that TYRP1 exhibits tyrosine hydroxylase, L-DOPA oxidase, dopachrome tautomerase, or DHICA (5,6-dihydroxyindole-2-carboxylic acid) oxidase activity [9,10,11]. However, purified recombinant intra-melanosomal human TYRP1 expressed in insect cells neither showed hydroxylase activity nor oxidase activity [4]. Unfortunately, dopachrome tautomerase activity could not be evaluated because of unavailability of suitable substrates. The absence of hydroxylase and oxidase activity is not surprising in light of the presence of two redox-inactive zinc ions instead of copper ions, as observed in the active site of tyrosinases. Yet, TYRP1 does bind typical tyrosinase substrates and inhibitors such as tyrosine, mimosine, kojic acid, and tropolone [4]. These compounds bind via aromatic stacking interactions with H381, and hydrogen bonding with S394, but they do not directly interact with the zinc ions. The aromatic hydroxy and keto-groups of tyrosine and the L-DOPA analogue L-mimosine are hydrogen-bonded to the water molecule, bridging the two zinc ions [4].

Of the various inhibitors we tested, phenylthiourea (PTU) was the most potent inhibitor of human tyrosinase (Figure 1). It binds to TYRP1 with its aromatic ring pointing outwards from the active site, blocking the entrance to the active site. The thiourea group did not interact with the binuclear metal site. In contrast, in *Ipomoea batatas* catechol oxidase (PDB entry 1BUG, [12]), another binuclear copper-containing enzyme with a very similar fold to tyrosinase, the PTU phenyl ring, occupied the same position as in TYRP1, but the thiourea sulfur atom was inserted between the two active site copper ions, coordinating both of them. One conspicuous difference between the two enzymes, besides the nature of the active site metal ions, was the presence of a Phe residue instead of a Val residue at position 391 (TYRP1 numbering). In catechol oxidase, the Phe residue provided stabilizing edge-to-face aromatic interactions with the phenyl ring of PTU, which cannot be given by Val.

An important question is whether the nature of the bound metal ions in the active site (copper in TYR, but zinc in TYRP1) affects the binding mode of PTU. The ionic radii of Cu^2+^ and Zn^2+^ ions are very similar (0.73 and 0.74 Å for Cu^2+^ and Zn^2+^, respectively [13]) and also their charge is the same. Comparing the binuclear zinc-binding site of TYRP1 with the binuclear copper-binding sites of tyrosinases, no differences in coordination geometry were observed. Of course, during the reaction catalyzed by tyrosinases, the copper ions switched between the Cu^2+^ and Cu^1+^ states, and this may have affected the affinity for and binding mode of PTU. However, with the currently available evidence, it appears that PTU was bound by hydrophobic interactions at the entrance of the active site, blocking access to the enzyme. In catechol oxidase, a nearby Phe side chain provided stabilizing edge-to-face aromatic interactions with the phenyl ring of PTU, thus allowing PTU to bind more deeply into the active site.

Superimposing the TYRP1-3M PTU structure on the human TYR model (see the Materials and Methods section and [1]) shows that PTU can be accommodated in the same position and orientation in the TYR active site as observed in TYRP1-3M. The strong inhibition by PTU also indicates that recognition of the substrate’s carboxylate group and interaction with any “substrate-guiding residues” in the second shell of the active site appears not to be essential [13]. Finally, the binding mode of PTU suggests that new types of inhibitors could be generated by replacing the PTU thiourea sulfur and nitrogen atoms, or the mimosine amino and carboxylate groups with an aromatic ring. Two similar aromatic ring-containing inhibitors have been reported for tyrosinase, viz., deoxyarbutin [14] and lucidone [15]. Thus, our crystal structure of PTU-inhibited TYRP1-3M enabled us to identify key residues in substrate binding, and provides a platform to design novel compounds targeting melanogenic proteins.

**Figure 4:**
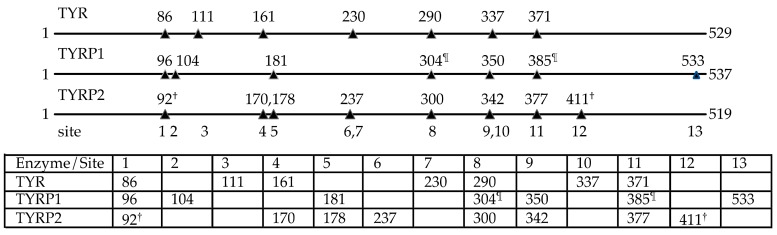
*N*-glycosylation sites in mammalian melanogenic enzymes. Indicated are the sequence positions of Asn residues that are part of N-X-S/T *N*-glycosylation sequence motifs in human, mouse, rabbit, and rat TYR, TYRP1, and TYRP2. Thirteen different putative *N*-glycosylation sites are present. The residue numbering is according to the sequence numbering of human TYR, human TYRP1, and human TYRP2, respectively. The sequences of human, mouse, rabbit, and rat TYR, TYRP1, and TYRP2 were retrieved from the UniProtKB protein knowledgebase (www.uniprot.org), with entry numbers P14679 (human TYR), P11344 (mouse TYR), G1SYA0 (rabbit TYR), D4A9G4 (rat TYR), P17643 (human TYRP1), P07147 (mouse TYRP1), G1SLB6 (rabbit TYRP1), D3ZH71 (rat TYRP1), P40126 (human TYRP2), P29812 (mouse TYRP2), and G1TFA4 (rabbit TYRP2). ^†^ Asn92 is not present in human TYRP2; Asn411 is only present in mouse TYRP2. ^¶^ Asn304 is not present in mouse and rat TYRP1; Asn385 is not present in rabbit TYRP1.

**Figure 5:**
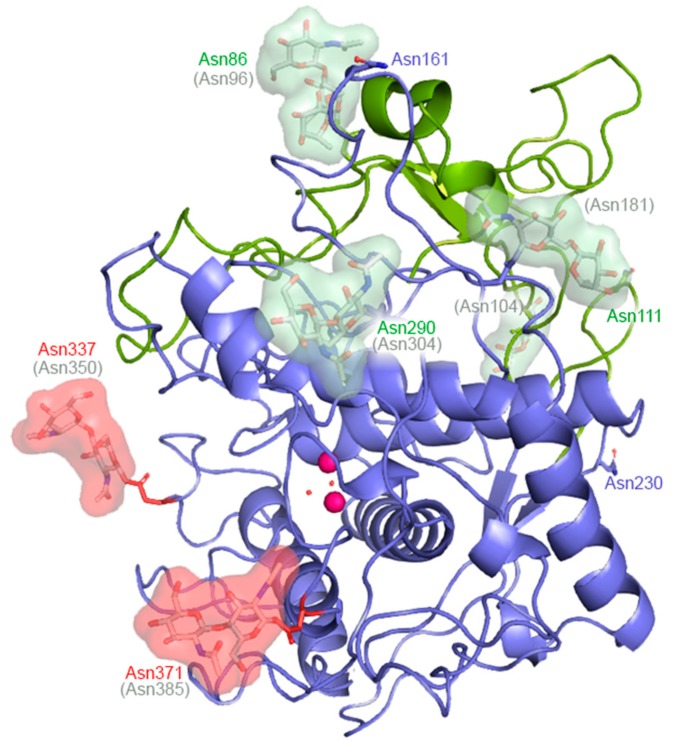
Location of the *N*-glycosylation sites in the model of human TYR and the crystal structure of glycosylated human TYRP1 (PDB 58ML [4]). The Asn residues of the *N*-glycosylation sequons (Asn-X-Ser/Thr) (Figure 4) in human TYR (in color) and human TYRP1 (in grey) are labelled. Glycans observed in the human TYRP1 crystal structure are depicted as sticks. Disease-associated Asn mutation sites are shown as red surfaces; the other *N*-glycosylation sites are shown as green surfaces. The tyrosinase-like domain is shown in blue and the Cys-rich domain in green. Metal ions are shown as pink spheres and coordinating waters are in red.

A further important question is whether differences in glycosylation may have consequences for activity. As summarized in Figure 4, the mammalian TYR, TYRP1, and TYRP2 proteins have distinct N-glycosylation patterns. For instance, all analyzed mammalian tyrosinases have N-glycosylation sites at Asn86, Asn111, Asn161, Asn230, Asn290, Asn337, and Asn 371 (human tyrosinase sequence numbering). In total, 13 distinct *N*-glycosylation positions are present in the three melanogenic enzymes, but none of them were fully conserved in all analyzed sequences (Figure 4). All *N*-glycosylation sites are on the surface of the protein models and fully accessible (Figure 5).

A search of the human genomic mutation database [16] revealed that no disease-related mutations are known for the *N*-glycosylation sequons of TYR Asn86, Asn111, Asn230, and Asn290, suggesting that glycosylation of these sites is not essential for functionality. These glycosylation sites are located at the back of the molecule when looking from the outside into the active site pocket, or behind peptide chains that prevent the glycan residues coming near the active site (Figure 5). A mutant protein (called D5 mutant), with these four asparagine residues replaced by Asps (plus an additional Asn337Asp mutation), still retained significant specific activity (reduced by only ≈30%) [17]. For Asn161, which is also located at the back of the protein, also no disease-related mutations are known. Adding the Asn161Asp mutation to the D5 mutant reduced the specific activity of this D6 mutant only moderately (by an additional 12%) [17]. This suggests that *N*-glycosylation at Asn86, Asn111, Asn161, Asn230, and Asn290 is not crucial for the functionality of human tyrosinase. On the other hand, mutations of the *N*-glycosylation sequons of TYR Asn337 and Asn371 have been found in patients with classic tyrosinase-dependent oculocutaneous albinism [18,19]. These *N*-glycosylation sites are located at the rim of the entrance to the active site pocket, about 15–20 Å from the active site copper ions, and the presence of glycan chains may affect the active site conformation (Figure 5). Although the Asn337Asp mutation was already included in the D5 mutant described above, adding the Asn371Asp mutation to the D5+Asn168Asp mutant dramatically reduced the specific activity (to 2% of that of the wild-type tyrosinase) [17]. This suggests that the Asn371 N-glycosylation site is the most important for the enzyme’s functionality, whether it be by stabilizing the enzyme, or by a direct effect on its activity, or by both. Further research is necessary to establish the precise role on Asn371 *N*-glycosylation.

## 4. Materials and Methods

### 4.1. Protein Expression and Purification

Overexpression and purification of TYR was done according to [20]. TYRP1-3M recombinant protein was prepared as described previously [4]. In brief, 2 L of sf21 cells were grown to a cell density of ≈1.0 million cells per mL before being infected with 2 mL recombinant baculovirus solution. The cells were cultured for 4 more days at 300 K until recombinant protein expression reached a plateau. The medium was clarified by centrifugation at 6000 g for 30 min and then concentrated to ≈100 mL using a 10 kDa cut-off QuixStand Benchtop System (GE Healthcare, Chicago, U.S.A.). The concentrated medium was incubated with 5 mL of Ni-NTA agarose resin (QIAGEN, Venlo, The Netherlands) for 20 min, which was then applied to a 20 mL gravity flow chromatography column (Econo-Pac column, Bio-Rad). The flow-through was discarded and the bound protein was washed with 100 mL of wash buffer (25 mM Tris-HCl, pH 7.8, 150 mM NaCl, 50 mM imidazole) and eluted with 20 mL of elution buffer (25 mM Tris-HCl, pH 7.8, 150 mM NaCl, 500 mM imidazole). The eluted protein solution was mixed with home-made TEV protease at a protein to TEV ratio of 50:1 (weight ratio) and dialyzed overnight at 277 K against dialysis buffer (25 mM Tris-HCl, pH 7.8, and 150 mM NaCl) to remove the C-terminal 6×His-tag. TEV protease and uncleaved protein were removed by Ni-NTA agarose (QIAGEN) chromatography. The flow-through containing the cleaved TYRP1-3M protein was collected, and the buffer was exchanged to ion-exchange buffer (25 mM Tris-HCl, pH 8.8, 50 mM NaCl) using a PD-10 desalting column (GE Healthcare). The resulting protein solution was then applied to a Mono Q 5/50 GL column (GE Healthcare) pre-equilibrated with the ion exchange buffer. The flow-through fractions that contain the pure TYRP1-3M were pooled, concentrated, and then applied to a final gel filtration chromatography column (Superdex 200 10/300 GL; GE Healthcare) pre-equilibrated with the crystallization buffer (10 mM Tris-HCl, pH 7.8, 100 mM NaCl). The elution fractions containing the pure protein were pooled and concentrated using a 30 kDa cut-off Amicon membrane (Merck Millipore, Burlington, Massachusetts, USA) to a concentration of ≈25 mg/mL, and stored at 193 K.

### 4.2. Activity Assays

Tyrosine hydroxylase and L-DOPA oxidase activities were determined at pH 6.5 and 298 K, as previously described [4]. On-plate tyrosinase activity was determined colorimetrically at pH 6.5 and 298 K in a Nunc Edge 96-well plate (Thermo Fisher Scientific, Waltham, Massachusetts, USA), in the presence of 0.4 μM L-tyrosine as substrate and 2 mM tropolone, 2 mM kojic acid, 2 mM mimosine, or 2 mM phenylthiourea as TYR inhibitors. Images were taken using a Photo HP Scanjet G3110 scanner (Hewlett-Packard, Palo Alto, California, USA) after 3 and 30 min.

### 4.3. Crystallization, Data Collection, and Processing

TYRP1-3M native crystals were generated in a condition containing 0.1 M Tris (pH 7.0), 0.2 M NaCl, and 30% (*w*/*v*) PEG 3000, with a protein to reservoir ratio of 3:1. Rod-like crystals appeared in 2 days and grew to full size in 7 days. For soaking PTU in the crystals, native crystals were fished in a cryo drop and soaked with reservoir solution supplemented with 20% (*v*/*v*) glycerol and saturated PTU for 5 to 10 min before flash freezing in liquid nitrogen for diffraction experiments.

X-ray diffraction data collection was carried out at the MASSIF-1 automatic beamline of the European Synchrotron (ESRF), Grenoble, France [21]. The dataset was processed and integrated using the program XDS [22] in combination with the program SCALA [23] from the CCP4 package [24]. The crystal belongs to P212121 space group. The structure was solved by molecular replacement in PHASER [25] using TYRP1 (PDB 5M8L) as a search model. The resulting single solution gave four molecules in the asymmetric unit, with clear difference density for PTU. Manual model rebuilding and refinement were iteratively performed with COOT [26] and PHENIX [27], respectively. Statistics of data collection and refinement are summarized in Table 1. Atomic coordinates and structure factor amplitudes have been deposited with the Protein Data Bank with entry code 5M8S.

### 4.4. Modeling

The full amino acid sequence of human tyrosinase (Uniprot entry P14679; 529 amino acid residues, including the N-terminal signal sequence and the C-terminal transmembrane and cytoplasmic domains) was submitted to the Swiss-model server [28]. Four models were generated by the server on the basis of the 5M8L (TYRP1 [4]) and 4YD9 (squid hemocyanin [29]) crystal structures present in the Protein Data Bank. The most complete model was that based on the TYRP1 structure (coverage of residues 19–455; 44.37% sequence identity) compared to coverage of residues 201-452 only in the models based on chains B, J, and M of squid hemocyanin (31.68%–33.50% sequence identity). Because 2 close contacts, 10 Ramachandran outliers, and 9 rotamer outliers were present in the TYRP1-based model, the model was energy-minimized using the Yasara server [30]. One Ramachandran outlier (Asp240) and seven poor rotamers remained, but several conserved side chains had acquired conformations that differed significantly from those in the TYRP1 structure. These inconsistencies were manually corrected using COOT [26], and the resulting model was energy-minimized using the ModRefiner server [31]. The final model retained one Ramachandran outlier (Ser331), but no poor rotamers. From a comparison of the various intermediate models, the errors in the modeled Ca positions were estimated to be around 0.5 Å. Because no 3D-structural information from homologous proteins was available for residues 455-529, the conformation of these residues was assessed using the PredictProtein [32] and Foldex [33] servers, as well as the JPRED4 server [34].

## Figures and Tables

**Table 1:** Statistics of data collection and refinement.

Data Collection	TYRP1-3M_PTU
Space group	*P*2_1_2_1_2_1_
Cell dimensions, a, b, c (Å)	90.1, 141.8, 191.7
Cell dimensions, α, β, γ (°)	90.0, 90.0, 90.0
Resolution	48.92–2.2 (2.32–2.2) ^1^
R_merge_ (%)	10.3 (84.7) ^1^
*I/σI*	10.0 (1.4) ^1^
Completeness (%)	99.4 (99.3) ^1^
Redundancy	4.0 (3.6) ^1^
**Refinement**	
Resolution (Å)	48.92–2.2
No. of reflections	124,119
*R*_work_/*R*_free_ (%)	19.36/23.49
No. of atoms	
Protein	14,216
Water molecules	722
Carbohydrate	635
Zinc ions	9
Ligand	40
*B* factors (Å^2^)	
Protein	43.485
Water	45.518
Carbohydrate	60.677
Zn	33.896
Ligand	40.008
**R.M.S. deviations**	
Bond lengths (Å)	0.009
Bond angles (°)	1.220
PDB entry	5M8S

^1^ Data between parentheses are for the highest resolution shell.

## Abbreviations

L-DOPA	L-3,4-dihydroxyphenylalanine
MBTH	3-methyl-2-benzothiazolinone hydrazine hydrochloride hydrate
PDB	Protein Data Bank (www.rcsb.org)
PTU	Phenylthiourea
TYR	Tyrosinase
TYRP1	Tyrosinase-related protein 1
TYRP1-3M	Triple (Y362F/R374S/T391V) mutant of TYRP1, with the three non-conserved active site residues replaced by the corresponding ones of TYR
TYRP2	Tyrosinase-related protein 2
